# Sex differences in DNA methylation across gestation: a large scale, cross-cohort, multi-tissue analysis

**DOI:** 10.1007/s00018-024-05208-0

**Published:** 2024-04-10

**Authors:** Darina Czamara, Linda Dieckmann, Marius Lahti-Pulkkinen, Cristiana Cruceanu, Wolfgang Henrich, Andreas Plagemann, Katri Räikkönen, Thorsten Braun, Elisabeth B. Binder, Jari Lahti, Sonja Entringer

**Affiliations:** 1 Department Genes and Environment, Max Planck Institute of Psychiatry, Munich, Germany; 2 International Max Planck Research School for Translational Psychiatry, Munich, Germany; 3 Department of Psychology and Logopedics, Faculty of Medicine, University of Helsinki, Helsinki, Finland; 4 Finnish Institute for Health and Welfare, Helsinki, Finland; 5 Centre for Cardiovascular Science, Queen’s Medical Research Institute, University of Edinburgh, Edinburgh, UK; 6 Department of Physiology and Pharmacology, Karolinska Institutet, Stockholm, Sweden; 7 Department of Obstetrics, Charité-Universitätsmedizin Berlin, Corporate Member of Freie Universität Berlin, Humboldt-Universität Zu Berlin, Berlin, Germany; 8 Department of Experimental Obstetrics, Charité-Universitätsmedizin Berlin, Corporate Member of Freie Universität Berlin, Humboldt-Universität Zu Berlin, Berlin, Germany; 9 Department of Obstetrics and Gynecology, HUS Helsinki University Hospital, Helsinki, Finland; 10 Department of Psychiatry and Behavioral Sciences, School of Medicine, Emory University, Atlanta, GA USA; 11 Institute of Medical Psychology, Charité-Universitätsmedizin Berlin, Corporate Member of Freie Universität Berlin, Humboldt-Universität Zu Berlin, Berlin, Germany; 12 Department of Pediatrics, Health and Disease Research Program, School of Medicine, University of California, Irvine, CA USA

**Keywords:** CVS, DNA methylation, Epigenetics, Placenta, Sex

## Abstract

Biological sex is a key variable influencing many physiological systems. Disease prevalence as well as treatment success can be modified by sex. Differences emerge already early in life and include pregnancy complications and adverse birth outcomes. The placenta is a critical organ for fetal development and shows sex-based differences in the expression of hormones and cytokines. Epigenetic regulation, such as DNA methylation (DNAm), may underlie the previously reported placental sexual dimorphism. We associated placental DNAm with fetal sex in three cohorts. Individual cohort results were meta-analyzed with random-effects modelling. CpG-sites differentially methylated with sex were further investigated regarding pathway enrichment, overlap with methylation quantitative trait loci (meQTLs), and hits from phenome-wide association studies (PheWAS). We evaluated the consistency of findings across tissues (CVS, i.e. chorionic villus sampling from early placenta, and cord blood) as well as with gene expression. We identified 10,320 epigenome-wide significant sex-differentially methylated probes (DMPs) spread throughout the epigenome of the placenta at birth. Most DMPs presented with lower DNAm levels in females. DMPs mapped to genes upregulated in brain, were enriched for neurodevelopmental pathways and significantly overlapped with meQTLs and PheWAS hits. Effect sizes were moderately correlated between CVS and placenta at birth, but only weakly correlated between birth placenta and cord blood. Sex differential gene expression in birth placenta was less pronounced and implicated genetic regions only marginally overlapped with those associated with differential DNAm. Our study provides an integrative perspective on sex-differential DNAm in perinatal tissues underscoring the possible link between placenta and brain.

**Supplementary Information:**

The online version contains supplementary material available at 10.1007/s00018-024-05208-0.

## Introduction

Biological sex as defined by the sex chromosome complement (i.e., XX or XY) is a key variable influencing many physiological systems [1]. Disease prevalence, disease progression as well as treatment success are modified by sex [2]. These observed differences between the sexes may emerge as a result from genetic and hormonal differences and from differential responses to and interactions with environmental factors, including infection, diet, drugs and stress [3]. Sex differences in disease prevalence emerge very early in life and include differences in pregnancy complications and adverse birth outcomes [4–6]. Furthermore, animal as well as human studies provide evidence for a diverse set of intrauterine exposures having differential effects on males and females [7–9].

The placenta is an essential organ for embryonic and fetal maturation. It provides nutrients, transfers respiratory gases and secretes hormones to ensure adequate fetal growth and development. The placenta receives and transduces signals to and from the pregnant mother’s and fetal compartments [10]. It is the conduit between the pregnant mother and fetal environment, and thus it plays a key role in mediating effects of exposures on fetal development and long-term disease risk [11]. The placental development and function are primarily regulated by the fetal genome. This is highlighted by the fact that most placental cells have the same sex chromosomes as the fetus [12]. The placenta shows sex-based differences in the expression of hormones and cytokines [13, 14], and at the level of the transcriptome [15]. Epigenetic regulation may underlie this observed placental sexual dimorphism [16]. Indeed, several studies have investigated the link between fetal sex and placental DNA methylation (DNAm) and identified sex differences in the methylation of CpG-sites in the placenta [17–21]. However, several of these studies are very limited in terms of sample size [18, 20, 21], included only placental tissue samples collected after birth [16, 18–20, 22], pooled data from several publicly available data sets [17, 19] or did not include comparisons of DNAm profiles of placental samples with other perinatal tissues [17].

In the current study, we aimed to overcome these limitations and investigated the effects of fetal sex on autosomal placental DNAm profiles at birth with a total of 746 placental samples across three cohorts covering 659,048 overlapping DNAm probes. Furthermore, in one cohort, DNAm profiles from first trimester chorionic villus sampling (CVS) were available providing the opportunity to assess which of the DNAm differences between male and female fetuses were consistent across gestation. Additionally, we had gene expression data in both CVS and birth placenta for a subset of samples, allowing us to investigate if changes in DNAm matched to those observed in gene expression profiles. Moreover, cross-tissue (CVS, birth placenta, cord blood) comparison of sex differences was evaluated in a sample of N = 65 individuals with all three tissues available. Furthermore, we checked if sexually dimorphic DNAm sites were associated with genetic variants acting as sex-specific or sex-consistent methylation quantitative trait loci (meQTLs), and evaluated possible overlap of these variants with significantly trait-associated single nucleotide polymorphisms (SNPs) from genome-wide association studies (GWAS) using phenome-wide association analysis (PheWAS). Our study provides an integrative perspective on sex-differential DNAm in perinatal tissues.

## Materials and methods

### Study populations

Our analysis included three cohorts: the betamethasone (BET) sample, the InTraUterine sampling in early pregnancy (ITU) sample and the Prediction and Prevention of Preeclampsia and Intrauterine Growth Restriction (PREDO) sample.

#### BET

The BET cohort is described in detail in [23–25]. In brief, pregnant mothers exposed to a single course of antenatal BET (Celestan^®^, MSD GmbH, Haar, Germany) for fetal lung maturation (2 × 12 mg intramuscular; *n* = 54) between 23 + 5 weeks to 34 + 0 weeks (weeks + days of gestation) were recruited prospectively before birth and compared to a gestational-age-matched control group that received no antenatal BET (*n* = 85). The control group consisted of gestational age (weeks) and fetal sex-matched pregnancies at the time of delivery. This study was approved by the Ethics Committee of the Charité Universitäts-medizin, Berlin, Germany.

#### ITU

The InTraUterine sampling in early pregnancy (ITU) is a prospective pregnancy cohort study [26]. It includes 943 pregnant Finnish women, their partners and their singleton children born alive between April 2012 and December 2017. Women were recruited through the national, voluntary trisomy 21 screening between 9 + 0 weeks and 21 + 6 gestational weeks. Of the participating women, 544 were screened positive and underwent fetal chromosomal testing. Test results then suggested no fetal chromosomal abnormality. Three hundred and ninety-nine women who screened negative and did not undergo fetal chromosomal testing were assessed as controls. The ITU research protocol has been approved by the Coordinating Ethics Committee of the Helsinki and Uusimaa Hospital District, Finland.

#### PREDO

The Prediction and Prevention of Preeclampsia and Intrauterine Growth Restriction (PREDO) study is a prospective, multicenter study of Finnish women who gave birth to a singleton live child between January 2006 and December 2010 [27]. The cohort includes 4774 mother–child dyads. PREDO recruited women when they visited antenatal clinics at any of the 10 study hospitals for their first ultrasound screening at 12 + 0 weeks to 13 + 6 weeks + days of gestation. Two groups of pregnant women were enrolled based on two eligibility criteria: pregnant women with a known clinical risk factor status for preeclampsia and intrauterine growth restriction (IUGR) and pregnant women who volunteered to participate regardless of their risk factor status for preeclampsia and IUGR. The first criterion was used to enrich the cohort for preeclampsia. The study protocol was approved by the Ethics Committee of Obstetrics and Gynaecology and Women, Children and Psychiatry of the Helsinki and Uusimaa Hospital District and by the participating hospitals.

#### Pregnancy and birth outcome data

Gestational age at sampling was based on fetal ultrasound. Child sex, maternal age at delivery and smoking during pregnancy (yes/no) were obtained from pre- and postnatal assessments and medical chart abstractions in the BET study, and extracted from the Finnish Medical Birth Register (MBR) [28] for ITU and PREDO. Further characteristics such as placental weight, birth weight, birth length and head circumference, mode of delivery (vaginal delivery or caesarean section), and induction of labor (yes or no) were obtained from medical records or the MBR.

### Biological sampling and pre-processing of omics data

#### Placental tissue sampling in BET, ITU and PREDO

In the BET study, full-thickness placental biopsies were taken by a uniform random sampling protocol from both peripheral and central areas starting immediately after delivery. In ITU, placenta samples were collected at birth, whereby midwives/trained staff took nine-site biopsies (within maximum 120 min after delivery) from the fetal side of the placenta. In PREDO, placenta samples were collected at birth, whereby midwives/trained staff took nine-site biopsies (within maximum 90 min after delivery) from the decidual side of the placenta. All samples were stored at − 80 °C.

#### Chorionic villus sampling (CVS) and cord blood sampling in ITU

In the ITU study also first-trimester placental biopsies as well as cord blood samples were available. First trimester placenta samples were obtained from leftover CVS, due to indications of elevated risk for chromosomal abnormalities between 10 and 15 weeks of gestation. Cord blood samples were taken immediately after birth by a midwife.

#### Pre-processing of DNA methylation (DNAm) in CVS, placenta and cordblood

DNA was extracted according to standard procedures and DNAm was assessed using the Illumina Infinium MethylationEPIC array v1 (Illumina, San Diego, USA). All DNAm data were pre-processed in the same way, as described in detail previously [29, 30] using an adapted pipeline from [31] implemented in the R package *minfi* [32]. Probes with SNPs at the CpG-site as well as probes reported to be cross-hybridizing [33, 34] were removed. Beta values were normalized using stratified quantile normalization [35] and beta-mixture quantile normalization (BMIQ) [36]. Batch-effects were removed performing *ComBat* [37] on the M-values (see Figure S1A–C for PCA-plots illustrating how much variability was removed due to Combat), and only autosomal DNAm probes were analyzed. The final datasets comprised 137 placenta samples (*n* = 708,222 probes) from the BET study, 264 CVS samples (*n* = 716,331 probes), 470 birth placental samples (*n* = 665,190 probes) and 426 cord blood samples (*n* = 724,075 probes) from ITU, and 139 placenta samples (*n* = 755,154 probes) from PREDO. Overall, 659,048 probes overlapped across all three birth placenta datasets.

#### Cell-type composition estimations from DNAm data

Cell-type composition into nucleated red blood cells, trophoblasts, syncytiotrophoblasts, stromal cells, Hofbauer and endothelial cells in CVS and placenta was estimated using the R package *planet*, by applying the robust partial correlation algorithm [38] based on a reference sample as described in [39]. Cell-type composition into nucleated red blood cells, granulocytes, monocytes, natural killer cells, B cells, CD4(+) T cells and CD8(+) T cells in cord blood was estimated in the R-package *minfi* [32] based on the approach proposed in [40].

#### Gene expression in CVS and placenta

The QuantSeq 3′ mRNA-Seq Library Prep Kit (Lexogen) was used to generate messenger RNA (mRNA) sequencing libraries from both CVS and birth placenta RNA samples in ITU. Libraries were multiplexed and sequenced using the Illumina HighSeq4000 system at a depth of 10 million reads per mRNA library. Adapter sequences were trimmed with *cutadapt* [41], sequenced reads were aligned to the human genome reference using the *STAR* aligner [42] and reads were quantified with *featureCounts* [43]. We filtered for autosomal genes with a raw read count of at least 10 in at least 90% of all individuals. This resulted in 260 individuals and 8754 genes for CVS and 478 individuals and 7955 genes for placenta.

#### Cell-type correction in gene expression analysis

We applied surrogate variable (SV) analysis to correct for possible batch effects and cell type heterogeneity in the ITU gene expression samples [37]. For CVS and placenta, the first SV was detected as significant (according to the permutation procedure implemented in the package) and used as covariate in the subsequent analyses.

#### Genotyping and imputation in cord blood and placenta

Genotyping was performed on Illumina Infinium Global Screening arrays for BET (from placenta) and ITU (from cord blood) and on Illumina Human Omni Express Arrays for PREDO (from cord blood). Genotypes were pre-processed separately for each cohort using a standard quality control pipeline—details are given in [29]. After quality control, each cohort was imputed based on the 1000 Genomes Phase 3 reference sample using *shapeit2* [44] and *impute2* [44]. After imputation, only SNPs with an info-score > 0.6 were retained in the analyses and converted into best-guessed genotypes using a probability threshold of 90%. We ran a second round of stringent quality control, keeping only SNPs with a call rate of at least 98%, a minor allele frequency of at least 5% and p-value for deviation from Hardy–Weinberg-Equilibrium > 1 × 10^–05^. This resulted in 136 individuals with 2,941,734 SNPs in BET, 425 individuals with 3,526,614 SNPs in ITU and 117 individuals with 5,284,432 SNPs in PREDO.

### Statistical analyses

If not stated otherwise, statistical analyses were conducted in R 4.2.1 (https://www.R-project.org/). All statistical analyses performed on DNAm data was based on M-values.

#### Robust linear regression on DNAm

We conducted robust linear regression models using White’s estimator as implemented in the R-package *MASS* in each cohort separately. We fitted one regression model per CpG using the M-value of the respective CpG-site as dependent variable and sex (using males as reference category) as predictor and gestational age at sampling, maternal smoking during pregnancy, maternal age as well as cell type proportions as covariates. The same analysis was conducted separately for each tissue in the ITU CVS (n = 264) and cord blood samples (n = 426) as well as in the ITU samples which had DNAm available in CVS, placenta at birth and cord blood (n = 65).

#### Meta-analysis

Inflation of test-statistics from the robust linear regression in each cohort was assessed using the R-package *bacon* [45]*.* Afterwards, we conducted a random-effects meta-analysis using DerSimonian-Laird random effects model as implemented in an extension of *METAL* [46] correcting the individual results for the estimated inflation-factors (BET: λ = 1.14, ITU: λ = 1.37, PREDO: λ = 1.17). As suggested by [47], we considered p-values below 9 × 10^–08^ as epigenome-wide significant in the meta-analyses. These CpGs were defined as differentially methylated probes (DMPs). We included those 758,101 CpG-sites in the meta-analysis which were present in at least one cohort, 711,597 CpG-sites were available in at least two cohorts. Manhattan plots were created using the R-package *GWASTools* [48].

#### Sensitivity analysis

To evaluate if any of the sex-differential DMPs were driven by cohort-specific variables such as maternal health, we performed sensitivity analyses. Herefore, we additionally covaried for betamethasone intake in the BET cohort (24/29 male/female with betamethasone treatment, 46/38 male/female without betamethasone treatment), prenatal testing (142/139 male/female with prenatal testing, 96/93 male/female without prenatal testing) and maternal education (45/38 male/female with primary or secondary education, 184/189 male/female with tertiary education) as proxy for socio-economic status (SES) in the ITU cohort as well as known risk factors for pre-eclampsia and IUGR (24/27 male/female with known risk, 43/45 male/female without known risk) and maternal education (available in 136 IDs with: 23/28 male/female with primary or secondary education, 42/43 male/female with tertiary education) in PREDO. Unfortunately, no SES measurement was available in the BET cohort. Maternal education was used as proxy for SES as data was most complete for this variable and maternal education has been shown to be highly correlated with SES [49]. Afterwards, we compared effect directions and effect sizes to the original estimates. For all DMPs, effect directions were consistent across analyses with and without further covariates. Furthermore, effect estimates correlated very highly across analyses (r > 0.93).

#### Differential methylated regions (DMRs)

We calculated DMRs with *comb-p* [50] using the meta-analysis p-value for each CpG-site, requiring a nominal significant p-value to define a region and setting a cut-off of 200 base pairs to extend a region if another CpG-site with an at least nominal significant p-value was found within that region. Afterwards, only regions presenting with an epigenome-wide significant p-value (p < 9 × 10^–08^) were retained.

#### Enrichment for genomic location

To check if DMPs were enriched for specific genomic locations, we mapped them with regards to relation to CpG-islands, based on Illumina’s annotation, as well as with regards to location in genes using the R-package *ChipSeeker* [51]. To test for enrichment, we applied two-sided Fisher-tests using those 747,781 CpGs which were included in our analysis but did not show epigenome-wide significance in the meta-analysis as background.

#### Enrichment for pathways and tissues

For enrichment analysis, we mapped CpG-sites to the nearest gene with the R-package *bumphunter* [52]. DMPs mapped to 3,817 unique genes. We performed enrichment tests for GO terms and tissue specific expression in the Genotype-Tissue Expression (GTEx) project v8 [53] with FUMA (https://fuma.ctglab.nl, [54]). All CpGs included in the analysis mapped to unique 20,214 genes and were used as background. Furthermore, we checked for tissue specific gene expression in the Human Protein Atlas [55] using the R-package *TissueEnrich* [56].

#### meQTL analysis in birth placenta

The datasets used for meQTL calculation comprised 136 individuals (70 males and 66 females) with 2,941,734 SNPs available in BET, 425 individuals (219 males and 206 females) with 3,526,614 SNPs in ITU and 117 individuals (57 males and 60 females) with 5,284,432 SNPs in PREDO. To test for enrichment with meQTLs (methylation quantitative trait loci), we first calculated meQTLs in each cohort using matrixEQTL [57] and a p-value threshold of 0.05. Within each cohort separately, M-values of each CpG-site were tested for association with SNP-genotypes in a 150 kb window around the specific CpG, using maternal smoking during pregnancy, gestational age, cell type composition, the first two multi-dimensional-scaling-components for population stratification as well as sex as covariates. Furthermore, we ran sex-stratified meQTL analyses in each cohort. Afterwards, we meta-analysed meQTL results in BET, ITU and PREDO as well as from the sex-stratified analyses with the DerSimonian-Laird random effects model as implemented in an extension of *METAL* [46]. Meta-analysis p-values below 1 × 10^–08^ were considered as significant *cis* meQTLs based on the threshold used in [58]. Only meQTLs where the specific SNP and the specific CpG were available in at least 2 of the 3 cohorts were considered further. This resulted in 3,040,103 significant CpG-SNP combinations in the overall sample (based on 678 individuals, formed by 1,087,708 unique SNPs and 70,855 unique CpGs), 1,634,651 significant *cis* meQTLs in males (based on 346 males, formed by 721,621 unique SNPs and 42,751 unique CpGs) and 1,461,207 significant *cis* meQTLs in females (based on 332 males, formed by 664,352 unique SNPs and 39,918 unique CpGs).

#### Sex-consistent and sex-specific meQTLs

In a next step, we wanted to evaluate if significant meQTLs were different between sexes. At first, we checked the 3,040,103 significant CpG-SNP combinations in the overall sample for direction of effects in the male-only and female-only samples. For the high majority (over 99%), the direction of effects was consistent in males and females. To evaluate sex-specific effects, we checked if the effect direction in the significant male-only meQTLs was consistent with effect direction in the female-only sample and vice versa. Again here, effect directions were consistent for over 99% of combinations. Based on these results, we created a list of sex-consistent and sex-specific SNP-CpG-combinations. Sex-consistent meQTLs were defined as SNP-CpG-combinations which were significant in the overall sample and showed the same direction of effects in males and females. Sex-specific meQTLs were defined as SNP-CpG-combinations which were significant in the male-only or female-only samples and showed a different effect direction in the respective different sex. Few CpGs (n = 167) were contained in both groups. To create two disjunct groups, the specific meQTLs were categorized into the group that presented with the lowest p-value. This resulted in 3,039,847 sex-consistent meQTLs (formed by 1,087,627 unique SNPs and 70,848 unique CpGs) and only 173 sex-specific meQTLs (formed by 169 unique SNPs and 22 unique CpGs).

#### Enrichment for meQTLs

We checked if the 10,320 epigenome-wide significant sex-differential DMPs overlapped with CpGs involved in sex-consistent and sex-specific meQTLs. To test if the overlap was significant, we applied Fisher-tests.

#### Phenome-wide association analysis (PheWAS)

To evaluate if SNPs implicated in meQTLs which overlapped with DMPs were also associated with other phenotypes, we performed a PheWAS. For each overlapping meQTL, we chose the SNP with the lowest p-value, yielding a list of 1924 unique SNPs. GWAS associations for these SNPs were extracted from the MRC IEU OpenGWAS platform (https://gwas.mrcieu.ac.uk). We focused on phenotypes originating from the batches UK Biobank study, the NHGRI-EBI GWAS Catalog, and a GWAS on brain imaging phenotypes based on UK Biobank data. This list was further reduced and filtered for duplicates and high similarity in trait names as described in Krontira et al. [59] resulting in a final list of 7503 phenotypes.

Extracted associations were corrected by false discovery rate (FDR, across phenotypes and SNPs). Overall, 12,035 associations (made up of 1150 unique SNPs and 1698 unique GWAS traits) survived multiple testing correction at FDR 0.05 and associated traits were manually grouped into the broad domain categories “anthropometric”, “blood-cell composition”, “bloodmarker”, “brain imaging”, “disease”, “education”, “environment”, “metabolism”, “nutrition”, “puberty” and “other” (if less than 1% of hits matched into the respective category). We applied the same approach on sex-stratified GWAS focusing on traits identified in [60]. These authors conducted a genome and trait-wide genetic effect comparison between males and females and identified 103 traits where at least one autosomal genetic variant presented with a significantly different effect at a p-value threshold of 1 × 10^−8^ threshold. Overall, 87 associations (made up of 41 unique SNPs and 41 unique GWAS traits) survived multiple testing correction at FDR 0.05. These associations grouped into the (manually created) broad domains “anthropometric”, “blood-cell composition”, “disease” and “metabolism”.

#### Differential gene expression

Differential gene expression with sex in ITU CVS and birth placenta was tested using the R-package *DESeq2* [61]. SV, gestational age at sampling and maternal smoking were used as covariates. For placental gene expression, we additionally covaried for induced labor yes/no and caesarian section yes/no. P-values were FDR-corrected at 0.05. The same analysis was conducted in the 91 individuals with RNASeq available in CVS and placenta.

## Results

We investigated sex-differential DNAm in birth placenta, correlation with sex-differential DNAm in other perinatal tissues and if implicated DNAm sites were genetically regulated by meQTLs. Our analysis included three cohorts: BET, ITU and PREDO. While the cohorts did not differ with regards to proportion of offspring sex, the BET cohort presented with lower maternal age and lower gestational age at birth, and hence also with lower birth weight and lower head circumference, as compared to ITU and PREDO. Furthermore, the prevalence of smoking during pregnancy was higher in BET compared to the other cohorts (see Table 1).

**Table 1:** Demographics of BET, ITU and PREDO

Phenotype	BET, n = 137	ITU, n = 470	PREDO, n = 139	p-value^a^
*Birth characteristics*				
Birth weight (kg), mean (SD)	3.15 (0.48)	3.54 (0.49)	3.43 (0.52)	**2.91 × 10**^**–14**^
Birth length (cm), mean (SD)	49.89 (2.65)	50.18 (2.25)	49.65 (2.53)	5.53 × 10^–02^
Gestational age at birth (days), mean (SD)	38.16 (1.95)	39.99 (1.55)	39.89 (1.43)	**9.04 × 10**^**–29**^
Head circumference (cm), mean (SD)	34.14 (1.56)	35.10 (1.55)	35.19 (1.34)	**1.02 × 10**^**–10**^
Placental weight (kg), mean (SD)	0.57 (0.12)	0.58 (0.12)	0.59 (0.11)	4.26 × 10^–01^
Preterm birth (< 37 weeks of gestation), %	34 (24.8%)	16 (3.4%)	5 (3.6%)	**5.75 × 10**^**–17**^
Sex male, %	70 (51.1%)	238 (50.6%)	67 (48.2%)	8.60 × 10^–01^
*Maternal characteristics*				
Maternal age (years), mean (SD)	29.19 (5.79)	34.51 (4.85)	32.04 (5.17)	**2.00 × 10**^**–25**^
Smoking during pregnancy yes, %	32 (23.4%)	11 (2.3%)	13 (9.5%)	**1.23 × 10**^**–15**^

^a^Nominal p-values are presented based on ANOVAs in case of quantitative variables and based on Chi-Square-tests in case of binary variables. P-values < 0.05 are depicted in bold

### Autosomal DNAm sex differences are wide-spread in the human placental epigenome with females showing hypomethylation

**Fig. 1:**
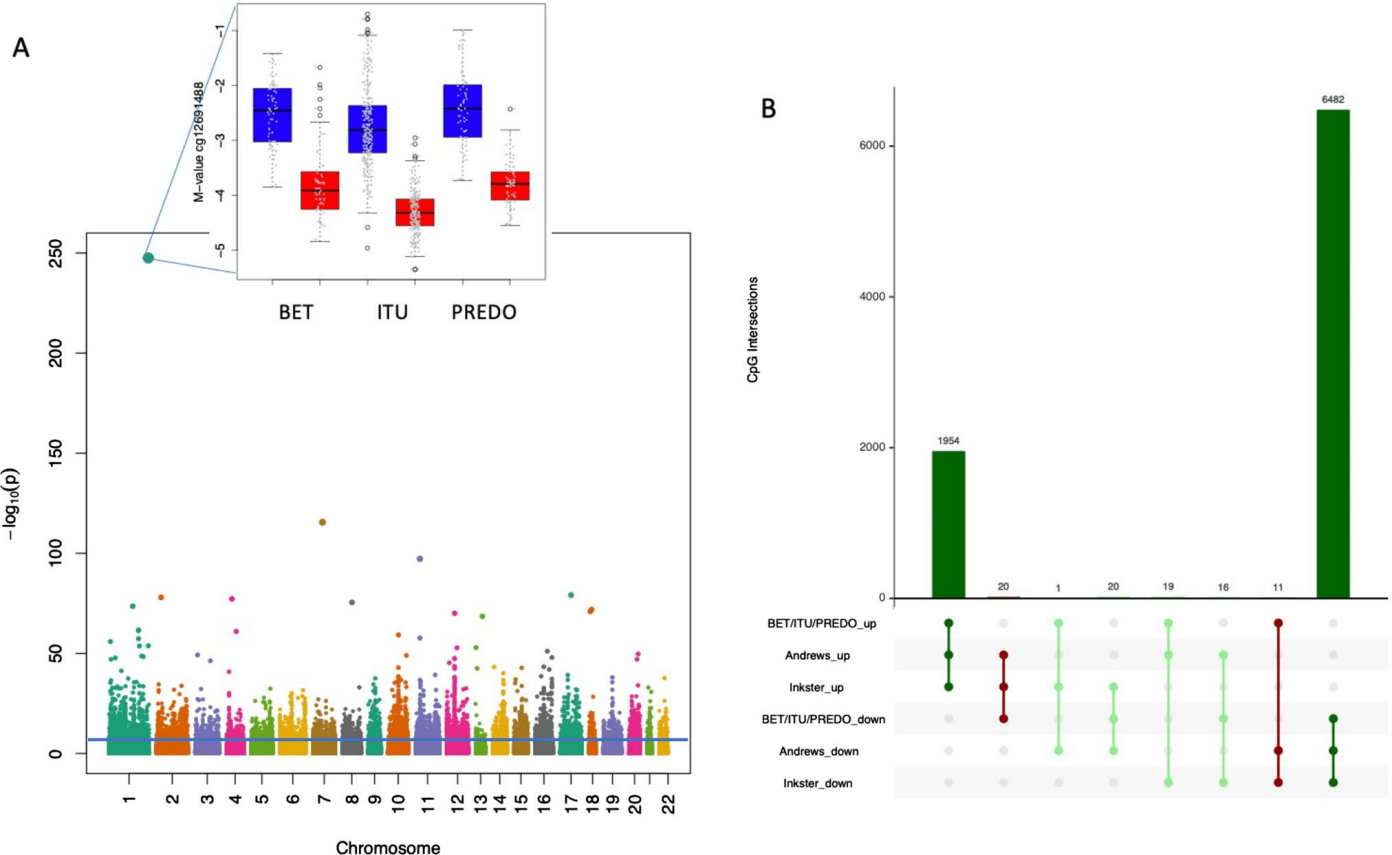
Manhattan plot of meta-analysis of differential DNAm and overlap with other studies. Manhattan plot of meta-analysis of association of sex and DNAm in placenta. The x-axis denotes the chromosomal position (based on hg19), the y-axis − log10 (p-values). The blue line indicates the p-value-threshold for epigenome-wide significance (p < 9 × 10^–08^). For cg12691488, the CpG-site with the lowest p-value, boxplots of M-values in males (blue) and females (red) are depicted for BET (left), ITU (middle) and PREDO (right) (**A**) The upset-plot shows the effect direction for the 8523 CpG-sites which are epigenome-wide significant in at least one of the three studies (BET/ITU/PREDO, Andrews et al., Inkster et al., 290,439 CpG-sites were available in all three studies). “up” indicates CpG-sites with significantly higher DNAm in females as compared to males, “down” indicates CpG-sites with significantly lower DNAm in females as compared to males. Consistent effect-directions in all three studies are depicted in dark green, consistent effect-directions between BET/ITU/PREDO and one other study are depicted in light green, CpG-sites with inconsistent effect-directions between BET/ITU/PREDO and the other studies are depicted in red (**B**)

Random-effects meta-analysis based on 375 male and 371 female placentas revealed 10,320 CpG-sites (differentially methylated probes, i.e. DMPs) with epigenome-wide significant p-values below 9 × 10^–08^ (see Table S1 for summary statistics of all epigenome-wide significant CpG-sites, full summary statistics is available at 10.6084/m9.figshare.25526911). DMPs were spread throughout the epigenome (see Fig. 1A) and the majority of DMPs (n = 8790 CpG-sites) presented with lower DNAm levels in females as compared to males. Sensitivity analyses revealed that DMPs were robustly associated with sex, even after correcting for cohort-specific phenotypes (betamethasone intake, maternal risk for pre-eclampsia and IUGR, prenatal testing, SES).

Only 3 DMPs overlapped with the 600 CpG-sites used in [39] to derive estimates for placenta cell-type proportions. We found no consistent significant differences in cell-type proportions between males and females across BET, ITU and PREDO. Furthermore, we identified 1199 epigenome-wide significant differentially methylated regions (DMRs), including between 2 and 17 CpGs (mean = 3 CpGs, also see Figure S2 and Table S2).

In the next step, we tested if our results were consistent with two other recent studies reporting differential DNAm by sex in human placenta [17, 19]. Both studies were based on 450 K methylation arrays, hence we restricted this part of the analysis to the 290,439 CpG-sites which overlapped between all three studies. Overall, 8523 CpG-sites were epigenome-wide significant in at least one of the three studies and effect directions were highly significant across the studies (see Fig. 1B and Table S3). A recent study [62] evaluated DNAm differences between males and females in 358 placentae using the EPIC array. The majority of their reported available top hits (48 of 50 CpGs available in our analysis) were also associated with sex in our meta-analysis (n = 46 CpGs with p < 1 × 10^–03^) and all of their hits showed consisted effect directions with our results.

### CpG-sites associated with sex differences are enriched for neurodevelopmental pathways and genes specifically enriched in brain

**Fig. 2:**
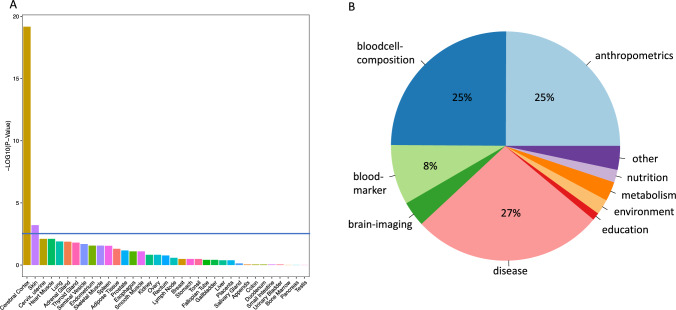
Tissue enrichment in the human protein atlas and PheWAS analysis. Tissue enrichment in the human protein atlas. The x-axis denotes the HPA-tissue, the y-axis − log10(p-values) for enrichment. The blue line indicates the p-value-threshold for FDR of 0.05 (**A**). Pie-chart of significant PheWAS-hits categories, based on 7503 phenotypes from the MRC IEU OpenGWAS platform. Domains associated with less than 1% of hits were grouped in the category “other”, for categories with more than 5% of hits proportions are labelled (**B**)

We investigated if DMPs mapped to specific regions or were enriched for specific pathways. Given that less than 50% of the DMPs were also available in [17] and [19], due to higher CpG-content on the EPIC array, we did not restrict the analysis to overlapping CpG-sites but included all 10,320 DMPs. DMPs were enriched in OpenSea (p = 3.54 × 10^–148^, OR = 1.71, Fisher-test, see Figure S3A) and distal intergenic regions (p = 9.74 × 10^–95^, OR = 1.53, Fisher-test, see Figure S3B). Pathway analysis based on 3,817 unique input genes (mapping to the DMPs by proximity) revealed enrichment in several pathways including keratinization and neurogenesis (see Tables S4–S6) and in a variety of transcription factor (TF) targets with highest enrichments for *ZNF596* and *PAX4*. Significant enrichments, albeit with a smaller amount of overlap, were also found for the glucocorticoid receptor (*GR*) as well as steroid hormone estrogen receptor (*ER*) but not for progesterone (*PR*) and androgen (*AR*, see Table S7). Based on gene expression in GTEx v8 [53], the 3,817 input genes were over-expressed across different tissues (see Table S8). The most significant enrichment was observed for genes specifically expressed in the brain with highest differential expression in cortical regions as well as hippocampus and hypothalamus. As no placenta specific gene expression is available in GTEx v8, we also used the Human Protein atlas [55]. While no enrichment for placenta-specific genes was detected, again here, the highest enrichment was found for brain, i.e. cerebral cortex (see Fig. 2A).

### CpG-sites associated with sex differences are enriched for sex-consistent meQTLs

We next conducted meQTL analyses combining all samples as well as using sex-stratified cohorts. At first, each cohort was analysed separately after which meQTL results were meta-analysed across all cohorts. Our results suggested that meQTLs were quite consistent across males and females: we identified 3,039,847 sex-consistent meQTLs (formed by 1,087,627 unique SNPs and 70,848 unique CpGs) at *cis*-significance level of 1 × 10^−08^ [58] and showing consistent effect directions in males and females. Of the DMPs, 2,162 (20.9%) overlapped with CpGs implicated in these sex-consistent meQTLs, i.e., meQTLs were significantly enriched for DMPs (p = 5.15 × 10^–280^, OR = 2.62, Fisher-test). In contrast, we could identify only 173 sex-specific meQTLs (formed by 169 unique SNPs and 22 unique CpGs) showing different effect directions between males and females (see methods for further information on the definition of sex-consistent and sex-specific meQTLs). None of these overlapped with the DMPs. The best SNP-CpG combination for the two meQTL groups, i.e., the combinations presenting with the lowest p-value for each CpG, are given in Tables S9 and S10.

### SNPs associated with DMPs are also GWAS hits in several domains

For each of the 2,162 DMPs which overlapped significantly with sex-consistent meQTLs, we chose the SNP with the lowest p-value. This yielded a list of 1924 unique SNPs. In a PheWAS focusing on these SNPs, 12,035 associations (made up of 1150 unique SNPs and 1698 GWAS traits) survived multiple testing correction. These associations, grouped by broad phenotype domain, are depicted in Fig. 2B. Overlaps with GWAS traits derived from the broad domains “disease” (27%, i.e. number of traits in this domain with regards to all significant traits including traits such as asthma and schizophrenia), “anthropometric” (25%, including traits such as standing height and weight), “blood-cell composition” (25%, including traits such as monocyte cell count and neutrophil cell count), “bloodmarker” (8%, including traits such as cholesterol levels and haemoglobin) and, to a lesser extent with “metabolism” and “brain imaging”. The exact grouping of GWAS traits to broad domains and number of associated meQTLs (overlapping with DMPs) is given in Table S11. Even though sex is usually corrected for in GWAS, we observed a high overlap with anthropometric traits inherently different between males and females, such as height. Therefore, we repeated the PheWAS in a more focused approach based on sex-stratified GWAS using 103 traits with significantly different genetic effect on males and females identified in [60]. Not all 7503 traits included in the first PheWAS, i.e. no brain imaging phenotypes, had been checked for sex-specific effects in the GWAS from Bernabeu et al. [60], hence the overlap between the two studies was limited. In the second PheWAS, 87 associations (made up of 41 unique SNPs and 41 GWAS traits) survived multiple testing correction. These associations grouped into the broad domains “anthropometric”, “blood-cell composition”, “disease” and “metabolism” (see Table S12). All of the significantly associated GWAS traits were also identified in the first, larger PheWAS using 7503 traits.

### CpG-sites associated with sex differences in term placenta show consistent effects in early-term placenta but not in cord blood

**Fig. 3:**
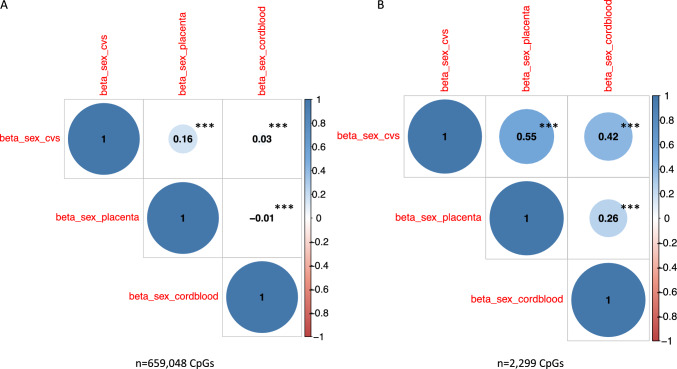
Cross-tissue correlation. Correlation-plot of effect sizes, i.e. beta weights of robust linear regression, of sex-differential DNAm analysis in ITU CVS tissue (n = 65 samples), ITU placenta tissue (n = 65) and ITU cordblood (n = 65). Overall, 659,048 CpG-sites overlapped between the tissues. All correlations were highly significant (p < 0.0001) and survived multiple-testing correction (**A**). Correlation-plot of effect sizes, i.e. beta weights of robust linear regression, of sex-differential DNAm analysis in ITU CVS tissue (n = 65 samples), ITU placenta tissue (n = 65) and ITU cordblood (n = 65), based on 2,299 CpG-sites significantly associated with sex in the 65 placental samples at FDR 0.05. All correlations were highly significant (p < 0.0001) and survived multiple-testing correction (**B**)

In the ITU cohort, not only placenta but also tissue from chorionic villus sampling (CVS) in early pregnancy as well as cord blood was analysed. We repeated the sex-differential DNAm analysis in all three tissues, focusing on those 65 individuals who had all three tissues available. The correlation of effect size estimates across all 659,048 CpGs which were available in all three tissues was weak (r = 0.16) between CVS and placenta and increased to moderate (r = 0.55) when focusing on the 2299 DMPs (at FDR 0.05, based on the 65 samples) in placenta only. In contrast to this, the correlation was weak (r = − 0.01) between placenta and cord blood and remained weak for the 2299 DMPs (r = 0.26, see Fig. 3A,B). A similar picture emerged when we used all available samples, regardless if they had all tissues available (all available CpGs: r = 0.39 CVS and placenta, r = 0.02 cord blood and placenta; 74,474 CpGs with FDR < 0.05 in placenta: r = 0.62 CVS and placenta, r = 0.10 cord blood and placenta).

### Few autosomal genes are sex-differentially expressed in placenta and these regions mostly do not overlap with differentially methylated CpG-sites

**Fig. 4:**
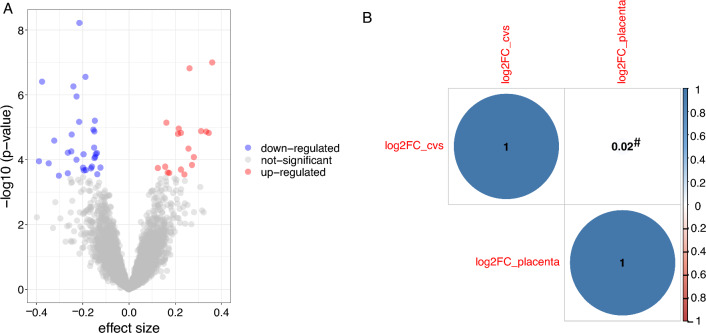
Volcano plot of differential gene expression and comparison to CVS gene expression. Volcano-plot of differential gene expression of sex in placenta. The x-axis denotes the effect-size, log2FoldChange, the y-axis − log10(p-values). FDR-significant associations where average gene expression is lower in females than in males are depicted in blue (“down-regulated”), FDR-significant associations where gene expression is higher in females than in males are depicted in red (“up-regulated”) (**A**). Correlation-plot of effect sizes (log2FoldChanges) of sex differential gene expression in ITU CVS tissue and ITU placenta tissue, based on 91 individuals and 7092 genes. The correlation was nominal significant (p < 0.05) (**B**)

Given the large amount of differentially methylated CpG-sites, we also checked for differential gene expression in birth placenta. Of the 7955 genes included in the analysis, 50 genes were differentially expressed with sex, with males and females showing up-regulation to a similar extent (see Fig. 4A and Table S13). Only 15 (30%) of these genes overlapped with genes mapping by proximity to DMPs, indicating only reduced overlap of differential DNAm and differential gene expression. We repeated the sex-differential gene expression analysis in CVS and placenta in those 91 individuals with both tissues. The correlation of effect size estimates across all 7092 autosomal genes which were available in both tissues was weak (r = 0.02) between CVS and placenta (see Fig. 4B).

Taken together, our analyses indicated wide-spread epigenome-wide sex differences in birth placenta which were mostly congruent with sex differences in DNAm in CVS, but not in cord blood tissue. MeQTLs for these DMPs significantly overlapped with GWAS hits.

## Discussion

In this study, we identified 10,320 placental DMPs, which were epigenome-wide significantly different between males and females. The majority of DMPs showed lower methylation in females. Genes mapping to DMPs where significantly enriched for neurodevelopmental pathways and for genes upregulated in brain tissue. Direction of effects were mostly congruent between birth placenta and CVS but not between birth placenta and cord blood tissue. MeQTLs significantly associated with the DMPs were associated with GWAS traits from several domains including anthropometrics and disease. Our results highlight the relevance of sex-specific methylation in placenta.

Sex-differential DNAm in placenta at birth is wide-spread in the human placental epigenome (see Fig. 1A), as has already been shown by other studies using 450 k arrays [17, 19, 62]. In our study, including 746 placental samples, we could replicate these findings for autosomal chromosomes, as well as identify additional differentially methylated sites on the EPIC array. As has already reported in the previous studies, females showed mostly hypomethylation compared to males.

The placenta is a crucial organ for fetal development and how it reacts to prenatal stress can also impact fetal brain development. It has been suggested, for instance, that neurobehavioral disorders are associated with placental disturbances and the relationship between the placenta and brain has been termed placenta-brain-axis [63]. Furthermore, sex-differences in the placenta are likely to be also involved in how the placental development is associated with fetal brain development [64]. This is supported by the fact that genes matching to epigenome-wide sex differential DNAm sites in our study were enriched for neuro-developmental pathways (see Table S4) and upregulated in cerebral cortex, hypothalamus and other brain tissues (see Fig. 2A and Table S8). This is in line with a study in mice showing that many genes involved in placental development also regulate the developing hypothalamus [65]. These enrichments underscore the hypothesis that sex differences in placenta might be implicated in brain maturation and development. This link is further supported by our PheWAS analysis. We found that meQTLs SNPs (SNPs that are regulating DNAm levels of placental sex-differential CpG-sites) significantly overlap with GWAS hits for brain imaging and mental health phenotypes, such as schizophrenia. While this requires replication in independent studies, it bares noting that a potential role of the placenta in the development of schizophrenia has already been described before [66].

The precise mechanisms underlying the differential methylation patterns observed in our and other studies between males and females that emerge very early in life are not yet fully understood but likely are based on genetic differences starting at conception when the oocyte and sperm cell fuse. This results in a difference in chromosome complement (i.e., an embryo carrying either XX or XY chromosomes), generating sex differences in the molecular makeup of all male and female cells [2]. The presence of two X chromosomes in females and only one in males requires the existence of a dosage compensation mechanism to ensure equal genetic contributions in both sexes [67]. However, due to random and unequal X inactivation across the X chromosome, gene expression levels on the X chromosome still differ between males and females [2, 67]. Thus, before gonadal formation occurs, and therefore before any sex-related hormonal dissimilarities appear, the differences in sex chromosome dosage can lead to a transcriptional sexual dimorphism that has been detected even before implantation [68]. The effect of X chromosome dosage and the presence of the Y chromosome and interaction between X and Y dosages may extend to transcription and methylation differences on the autosome [16, 67, 69, 70]. Furthermore, glucose and protein metabolism as well as mitochondrial function have been reported to differ between male and female fetuses, potentially also contributing to differential DNAm patterns [68]. Another mechanism that may underly sex-specific gene expression is genomic imprinting. Genomic imprinting is a form of epigenetic modification in which gene expression differs in an allele-specific manner depending on parent of origin [71, 72]. The differential expression of these imprinted genes can lead to distinct molecular and physiological characteristics in the placenta of male and female embryos.

DNAm profiles after delivery reflect the cumulative effects of exposures across gestation. It is controversial to what degree differences in gonadal hormone concentrations between male and female fetuses could contribute to the observed effects on DNAm patterns. Differences in androgens based on fetal sex were observed only during weeks 8–18 of gestation [73]. Inkster et al. [19] found no association of placental differentially methylated sites based on sex with nearby estrogen receptor (*ER)* or androgen* (AR*) binding sites. Furthermore, results of a study in adults investigating leucocyte DNAm suggest that global differences in autosomal DNA methylation are not driven by sex hormones [74]. However, in our findings, sex differential CpG-sites significantly overlapped with transcription factor binding sites of *ER* but not *AR* (see Table S7). Further studies across different tissues are needed to replicate or refute these findings. In our results, one of the strongest enrichments was found for transcription factor binding sites of *PAX4*, which plays a critical role in fetal development [75].

Within the ITU cohort, we were able to also investigate early placenta (CVS) as well as cord blood tissue. While we found very consistent effect directions of sex differential DNAm between CVS and placenta, only about 50% of our top hits showed the same effect direction also in cord blood. This is concordant to a recent study [62] which also reported tissue-specific DNAm differences between males and females. From these findings we conclude that sex-specific DNAm patterns emerge already quite early in pregnancy but do not necessarily persist across tissues which has already been observed by Bozack et al. [18]. Interestingly, the correlation of effect sizes of sex differential DNAm in placenta and cord blood increased from r = − 0.01 using all CpG-sites to r = 0.26 when focusing only on sex differential CpG-sites identified in placenta. This could indicate that a common, underlying cross-tissue regulation along the same pathways might be present for the CpG-sites with the strongest associations. This is supported by the fact that enrichment for neurodevelopmental gene ontology terms, that we found for sex differential CpG-sites in placenta, has also been reported for sex differential CpG-sites in cord blood samples [70, 76].

We observed wide-spread sex differences in placenta DNAm. Interestingly, even though DNAm can have consequences on gene expression, sex differences in gene expression were far less pronounced and the genetic locations of differentially expressed genes and differentially methylated CpG-sites did not fully overlap. These findings indicate that implicated DMPs are not necessarily also eQTMs (expression quantitative trait methylation sites), i.e. CpG-sites associated with changes in gene expression. Further studies are required to identify placental eQTM, based on CpG-sites included on the EPIC array, to assess if DMPs identified in our study are associated with gene expression, and to investigate if eQTMs are also sex-specific.

Furthermore, in our analysis, we corrected for two major delivery variables which have been associated with placental gene expression at birth: delivery mode (i.e. C-section) [77] and induced labor [78]. The fact that we did not observe much remaining variability in placental gene expression after this correction could also indicate that gene expression in placenta at birth represents the state and circumstances of birth rather than any developmental processes.

Finally, limitations of our study should be considered. Even though we used the same pre-processing protocol for DNAm across all cohorts and implemented a random-effects model to account for variability of effects across studies, it should be noted that each cohort was based on a different recruitment strategy and sampling protocol (i.e., fetal or decidual sampling of placental tissue). We additionally covaried for possible cohort-specific confounders such as betamethasone intake, prenatal testing or maternal risk for pre-eclampsia. These sensitivity analyses showed that the identified DMPs were very robustly associated with sex. Nevertheless, future studies are needed to investigate more carefully, if and how the placental sampling method, as well as maternal characteristics are associated with sex-differential DNAm in the placenta. Furthermore, sex-specific as well as sex-consistent meQTLs should also be investigated in more depth in larger studies, ideally also on single-cell resolution.

As shown in [29], placental sampling is highly associated with cell type composition. Even though we corrected for cell types using deconvolution methods which are standard in the field, these methods can only give an estimation of the underlying cell type proportions. Furthermore, they are limited to the cell-types which their reference samples are based on. While the R-package *planet* derives six major placental cell-types, other studies have found up to 29 different placental cell subtypes [79, 80]. Cell-type deconvolution methods work well for correction for cell-type variability but we cannot fully investigate if sex differential DNAm is consistent across different cell types or driven by different proportions of specific cell types in our study samples. Single-cell RNA-sequencing studies such as [72] are needed to answer these questions.

In addition, we here only focused on DNAm assessed on EPIC arrays. Sex could also have an impact on placental function through other epigenetic characteristics, e.g., hydroxymethylation [81], i.e. the replacement of the hydrogen atom at the C5 position in the cytosine with a hydroxymethyl-group, or histone modifications, e.g. acetylation [82]. These should be assessed in more detail in future studies.

We investigated sex-differential DNAm in perinatal tissues, identifying wide-spread differences in early placental DNAm and placenta at birth. We observed moderate correlations of sex-differential effects across gestation in placental tissue but only part of these effects were still retained in cord blood. The enrichment of placental DMPs with neurodevelopmental pathways and genes upregulated in the brain underpin the potential link between placenta and brain development. The high number of differentially methylated sites underscores the call for sex-stratified analysis also in DNAm studies.

The sex-related differences in placental DNA methylation patterns and, to a lower extent, gene expression, imply that male and female embryonic and fetal genomes may react quite differently to environmental exposures, which provides the molecular basis for sex-specific long-term effects originating during intrauterine environment [68].

### Supplementary Information

Below is the link to the electronic supplementary material.

Supplementary file1 (PDF 134 KB)

Supplementary file2 (PDF 314 KB)

Supplementary file3 (PDF 137 KB)

Supplementary file4 (PDF 320 KB)

Supplementary file5 (PDF 60 KB)

Supplementary file6 (XLSX 7192 KB)

## Data Availability

Due to the sensitive nature of the patient data used in the current study, the data sets are not and cannot be made publicly available. However, an interested researcher can obtain a de-identified data set after approval from the respective Study Board. Data requests of PREDO and ITU studies may be additionally subject to further review by the national register authorities and by the ethical committees. The code to the main analyses is publicly available at https://github.com/darinacz/DNAm_sex_placenta.

## Abbreviations

BET: Betamethasone sample
BMIQ: Beta-mixture quantile normalization
CVS: Chorionic villus sampling
DMPs: Differentially methylated probes
DMRs: Differential methylated regions
DNAm: DNA methylation
eQTM: Expression quantitative trait methylation site
FDR: False discovery rate
GTEx: Genotype-tissue expression
GWAS: Genome-wide association studies
ITU: InTraUterine sampling in early pregnancy sample
IUGR: Intrauterine growth restriction
MBR: Medical birth register
meQTLs: Methylation quantitative trait loci
PheWAS: Phenome-wide association studies
PREDO: Prediction and prevention of preeclampsia and intrauterine growth restriction sample
SES: Socio-economic status
SNP: Single nucleotide polymorphism
SV: Surrogate variable

## References

[1] Bale TL, Epperson CN (2017). Sex as a biological variable: who, what, when, why, and how. Neuropsychopharmacology.

[2] Mauvais-Jarvis F, Bairey Merz N, Barnes PJ, Brinton RD, Carrero JJ, DeMeo DL (2020). Sex and gender: modifiers of health, disease, and medicine. Lancet.

[3] Gabory A, Roseboom TJ, Moore T, Moore LG, Junien C (2013). Placental contribution to the origins of sexual dimorphism in health and diseases: sex chromosomes and epigenetics. Biol Sex Differ.

[4] Challis J, Newnham J, Petraglia F, Yeganegi M, Bocking A (2013). Fetal sex and preterm birth. Placenta.

[5] Peelen MJ, Kazemier BM, Ravelli AC, De Groot CJ, Van Der Post JA, Mol BW (2016). Impact of fetal gender on the risk of preterm birth, a national cohort study. Acta Obstet Gynecol Scand.

[6] Broere-Brown ZA, Adank MC, Benschop L, Tielemans M, Muka T, Goncalves R (2020). Fetal sex and maternal pregnancy outcomes: a systematic review and meta-analysis. Biol Sex Differ.

[7] DiPietro JA, Voegtline KM (2017). The gestational foundation of sex differences in development and vulnerability. Neuroscience.

[8] Franko KL, Forhead AJ, Fowden AL (2010). Differential effects of prenatal stress and glucocorticoid administration on postnatal growth and glucose metabolism in rats. J Endocrinol.

[9] Graham AM, Rasmussen JM, Entringer S, Ben Ward E, Rudolph MD, Gilmore JH (2019). Maternal cortisol concentrations during pregnancy and sex-specific associations with neonatal amygdala connectivity and emerging internalizing behaviors. Biol Psychiatry.

[10] Nugent BM, Bale TL (2015). The omniscient placenta: metabolic and epigenetic regulation of fetal programming. Front Neuroendocrinol.

[11] Braun T, Challis JR, Newnham JP, Sloboda DM (2013). Early-life glucocorticoid exposure: the hypothalamic-pituitary-adrenal axis, placental function, and long-term disease risk. Endocr Rev.

[12] Phuthong S, Reyes-Hernandez CG, Rodriguez-Rodriguez P, Ramiro-Cortijo D, Gil-Ortega M, Gonzalez-Blazquez R (2020). Sex differences in placental protein expression and efficiency in a rat model of fetal programming induced by maternal undernutrition. Int J Mol Sci.

[13] Mitchell AM, Palettas M, Christian LM (2017). Fetal sex is associated with maternal stimulated cytokine production, but not serum cytokine levels, in human pregnancy. Brain Behav Immun.

[14] Meakin AS, Cuffe JSM, Darby JRT, Morrison JL, Clifton VL (2021). Let's talk about placental sex, baby: understanding mechanisms that drive female- and male-specific fetal growth and developmental outcomes. Int J Mol Sci.

[15] Gonzalez TL, Sun T, Koeppel AF, Lee B, Wang ET, Farber CR (2018). Sex differences in the late first trimester human placenta transcriptome. Biol Sex Differ.

[16] Martin E, Smeester L, Bommarito PA, Grace MR, Boggess K, Kuban K (2017). Sexual epigenetic dimorphism in the human placenta: implications for susceptibility during the prenatal period. Epigenomics.

[17] Andrews SV, Yang IJ, Froehlich K, Oskotsky T, Sirota M (2022). Large-scale placenta DNA methylation integrated analysis reveals fetal sex-specific differentially methylated CpG sites and regions. Sci Rep.

[18] Bozack AK, Colicino E, Just AC, Wright RO, Baccarelli AA, Wright RJ (2022). Associations between infant sex and DNA methylation across umbilical cord blood, artery, and placenta samples. Epigenetics.

[19] Inkster AM, Yuan V, Konwar C, Matthews AM, Brown CJ, Robinson WP (2021). A cross-cohort analysis of autosomal DNA methylation sex differences in the term placenta. Biol Sex Differ.

[20] Cotton AM, Avila L, Penaherrera MS, Affleck JG, Robinson WP, Brown CJ (2009). Inactive X chromosome-specific reduction in placental DNA methylation. Hum Mol Genet.

[21] Gong S, Johnson MD, Dopierala J, Gaccioli F, Sovio U, Constancia M (2018). Genome-wide oxidative bisulfite sequencing identifies sex-specific methylation differences in the human placenta. Epigenetics.

[22] Gong S, Gaccioli F, Dopierala J, Sovio U, Cook E, Volders PJ (2021). The RNA landscape of the human placenta in health and disease. Nat Commun.

[23] Braun T, Husar A, Challis JR, Dudenhausen JW, Henrich W, Plagemann A (2013). Growth restricting effects of a single course of antenatal betamethasone treatment and the role of human placental lactogen. Placenta.

[24] Braun F, Hardt AK, Ehrlich L, Sloboda DM, Challis JRG, Plagemann A (2018). Sex-specific and lasting effects of a single course of antenatal betamethasone treatment on human placental 11beta-HSD2. Placenta.

[25] Czamara D, Dieckmann L, Roh S, Kraemer S, Rancourt RC, Sammallahti S (2021). Betamethasone administration during pregnancy is associated with placental epigenetic changes with implications for inflammation. Clin Epigenet.

[26] Kvist T, Sammallahti S, Lahti-Pulkkinen M, Cruceanu C, Czamara D, Dieckmann L (2022). Cohort profile: InTraUterine sampling in early pregnancy (ITU), a prospective pregnancy cohort study in Finland: study design and baseline characteristics. BMJ Open.

[27] Girchenko P, Lahti M, Tuovinen S, Savolainen K, Lahti J, Binder EB (2017). Cohort Profile: prediction and prevention of preeclampsia and intrauterine growth restriction (PREDO) study. Int J Epidemiol.

[28] Gissler M, Shelley J (2002). Quality of data on subsequent events in a routine Medical Birth Register. Med Inform Internet Med.

[29] Dieckmann L, Cruceanu C, Lahti-Pulkkinen M, Lahti J, Kvist T, Laivuori H (2022). Reliability of a novel approach for reference-based cell type estimation in human placental DNA methylation studies. Cell Mol Life Sci.

[30] Dieckmann L, Lahti-Pulkkinen M, Kvist T, Lahti J, DeWitt PE, Cruceanu C (2021). Characteristics of epigenetic aging across gestational and perinatal tissues. Clin Epigenetics.

[31] Maksimovic J, Phipson B, Oshlack A (2016). A cross-package Bioconductor workflow for analysing methylation array data. F1000Res.

[32] Aryee MJ, Jaffe AE, Corrada-Bravo H, Ladd-Acosta C, Feinberg AP, Hansen KD (2014). Minfi: a flexible and comprehensive Bioconductor package for the analysis of Infinium DNA methylation microarrays. Bioinformatics.

[33] Chen YA, Lemire M, Choufani S, Butcher DT, Grafodatskaya D, Zanke BW (2013). Discovery of cross-reactive probes and polymorphic CpGs in the Illumina Infinium HumanMethylation450 microarray. Epigenetics.

[34] McCartney DL, Walker RM, Morris SW, McIntosh AM, Porteous DJ, Evans KL (2016). Identification of polymorphic and off-target probe binding sites on the Illumina Infinium MethylationEPIC BeadChip. Genom Data.

[35] Touleimat N, Tost J (2012). Complete pipeline for Infinium((R)) Human Methylation 450K BeadChip data processing using subset quantile normalization for accurate DNA methylation estimation. Epigenomics.

[36] Teschendorff AE, Marabita F, Lechner M, Bartlett T, Tegner J, Gomez-Cabrero D (2013). A beta-mixture quantile normalization method for correcting probe design bias in Illumina Infinium 450 k DNA methylation data. Bioinformatics.

[37] Leek JT, Johnson WE, Parker HS, Jaffe AE, Storey JD (2012). The sva package for removing batch effects and other unwanted variation in high-throughput experiments. Bioinformatics.

[38] Teschendorff AE, Breeze CE, Zheng SC, Beck S (2017). A comparison of reference-based algorithms for correcting cell-type heterogeneity in Epigenome-Wide Association Studies. BMC Bioinformatics.

[39] Yuan V, Hui D, Yin Y, Penaherrera MS, Beristain AG, Robinson WP (2021). Cell-specific characterization of the placental methylome. BMC Genomics.

[40] Gervin K, Salas LA, Bakulski KM, van Zelm MC, Koestler DC, Wiencke JK (2019). Systematic evaluation and validation of reference and library selection methods for deconvolution of cord blood DNA methylation data. Clin Epigenet.

[41] Marcel M (2011). Cutadapt removes adapter sequences from high-throughput sequencing reads. EMBnet J.

[42] Dobin A, Davis CA, Schlesinger F, Drenkow J, Zaleski C, Jha S (2013). STAR: ultrafast universal RNA-seq aligner. Bioinformatics.

[43] Liao Y, Smyth GK, Shi W (2014). featureCounts: an efficient general purpose program for assigning sequence reads to genomic features. Bioinformatics.

[44] Delaneau O, Marchini J, Zagury JF (2011). A linear complexity phasing method for thousands of genomes. Nat Methods.

[45] van Iterson M, van Zwet EW, Consortium B, Heijmans BT (2017). Controlling bias and inflation in epigenome- and transcriptome-wide association studies using the empirical null distribution. Genome Biol.

[46] Willer CJ, Li Y, Abecasis GR (2010). METAL: fast and efficient meta-analysis of genomewide association scans. Bioinformatics.

[47] Mansell G, Gorrie-Stone TJ, Bao Y, Kumari M, Schalkwyk LS, Mill J (2019). Guidance for DNA methylation studies: statistical insights from the Illumina EPIC array. BMC Genom.

[48] Gogarten SM, Bhangale T, Conomos MP, Laurie CA, McHugh CP, Painter I (2012). GWASTools: an R/Bioconductor package for quality control and analysis of genome-wide association studies. Bioinformatics.

[49] Airikka A, Lahti-Pulkkinen M, Tuovinen S, Heinonen K, Lahti J, Girchenko P (2023). Maternal exposure to childhood maltreatment and mental and behavioral disorders in children. Eur Child Adolesc Psychiatry.

[50] Pedersen BS, Schwartz DA, Yang IV, Kechris KJ (2012). Comb-p: software for combining, analyzing, grouping and correcting spatially correlated P-values. Bioinformatics.

[51] Yu G, Wang LG, He QY (2015). ChIPseeker: an R/Bioconductor package for ChIP peak annotation, comparison and visualization. Bioinformatics.

[52] Jaffe AE, Murakami P, Lee H, Leek JT, Fallin MD, Feinberg AP (2012). Bump hunting to identify differentially methylated regions in epigenetic epidemiology studies. Int J Epidemiol.

[53] Consortium GT (2020). The GTEx Consortium atlas of genetic regulatory effects across human tissues. Science.

[54] Watanabe K, Taskesen E, van Bochoven A, Posthuma D (2017). Functional mapping and annotation of genetic associations with FUMA. Nat Commun.

[55] Uhlen M, Fagerberg L, Hallstrom BM, Lindskog C, Oksvold P, Mardinoglu A (2015). Proteomics. Tissue-based map of the human proteome. Science.

[56] Jain A, Tuteja G (2019). TissueEnrich: tissue-specific gene enrichment analysis. Bioinformatics.

[57] Shabalin AA (2012). Matrix eQTL: ultra fast eQTL analysis via large matrix operations. Bioinformatics.

[58] Min JL, Hemani G, Hannon E, Dekkers KF, Castillo-Fernandez J, Luijk R (2021). Genomic and phenotypic insights from an atlas of genetic effects on DNA methylation. Nat Genet.

[59] Krontira AC, Cruceanu C, Dony L, Kyrousi C, Link MH, Rek N et al (2024) Human cortical neurogenesis is altered via glucocorticoid-mediated regulation of ZBTB16 expression. Neuron 2024 Feb 27:S0896-6273(24)00089-8. 10.1016/j.neuron.2024.02.005 38442714

[60] Bernabeu E, Canela-Xandri O, Rawlik K, Talenti A, Prendergast J, Tenesa A (2021). Sex differences in genetic architecture in the UK Biobank. Nat Genet.

[61] Love MI, Huber W, Anders S (2014). Moderated estimation of fold change and dispersion for RNA-seq data with DESeq2. Genome Biol.

[62] Santos HP, Enggasser AE, Clark J, Roell K, Zhabotynsky V, Gower WA (2023). Sexually dimorphic methylation patterns characterize the placenta and blood from extremely preterm newborns. BMC Biol.

[63] Rosenfeld CS (2021). The placenta-brain-axis. J Neurosci Res.

[64] Bale TL (2016). The placenta and neurodevelopment: sex differences in prenatal vulnerability. Dialogues Clin Neurosci.

[65] Broad KD, Keverne EB (2011). Placental protection of the fetal brain during short-term food deprivation. Proc Natl Acad Sci USA.

[66] Ursini G, Punzi G, Chen Q, Marenco S, Robinson JF, Porcelli A (2018). Convergence of placenta biology and genetic risk for schizophrenia. Nat Med.

[67] Sharma A, Jamil MA, Nuesgen N, Schreiner F, Priebe L, Hoffmann P (2015). DNA methylation signature in peripheral blood reveals distinct characteristics of human X chromosome numerical aberrations. Clin Epigenetics.

[68] Bermejo-Alvarez P, Rizos D, Lonergan P, Gutierrez-Adan A (2011). Transcriptional sexual dimorphism during preimplantation embryo development and its consequences for developmental competence and adult health and disease. Reproduction.

[69] McCarthy NS, Melton PE, Cadby G, Yazar S, Franchina M, Moses EK (2014). Meta-analysis of human methylation data for evidence of sex-specific autosomal patterns. BMC Genomics.

[70] Maschietto M, Bastos LC, Tahira AC, Bastos EP, Euclydes VL, Brentani A (2017). Sex differences in DNA methylation of the cord blood are related to sex-bias psychiatric diseases. Sci Rep.

[71] Bartolomei MS, Ferguson-Smith AC (2011). Mammalian genomic imprinting. Cold Spring Harb Perspect Biol.

[72] Pilvar D, Reiman M, Pilvar A, Laan M (2019). Parent-of-origin-specific allelic expression in the human placenta is limited to established imprinted loci and it is stably maintained across pregnancy. Clin Epigenet.

[73] Hill M, Paskova A, Kanceva R, Velikova M, Kubatova J, Kancheva L (2014). Steroid profiling in pregnancy: a focus on the human fetus. J Steroid Biochem Mol Biol.

[74] Iwasaki M, Ono H, Kuchiba A, Kasuga Y, Yokoyama S, Onuma H (2012). Association of postmenopausal endogenous sex hormones with global methylation level of leukocyte DNA among Japanese women. BMC Cancer.

[75] Lorenzo PI, Juarez-Vicente F, Cobo-Vuilleumier N, Garcia-Dominguez M, Gauthier BR (2017). The diabetes-linked transcription factor PAX4: from gene to functional consequences. Genes (Basel)..

[76] Yousefi P, Huen K, Dave V, Barcellos L, Eskenazi B, Holland N (2015). Sex differences in DNA methylation assessed by 450 K BeadChip in newborns. BMC Genom.

[77] Kothiyal P, Schulkers K, Liu X, Hazrati S, Vilboux T, Gomez LM (2020). Differences in maternal gene expression in Cesarean section delivery compared with vaginal delivery. Sci Rep.

[78] Lee KJ, Shim SH, Kang KM, Kang JH, Park DY, Kim SH (2010). Global gene expression changes induced in the human placenta during labor. Placenta.

[79] Campbell KA, Colacino JA, Puttabyatappa M, Dou JF, Elkin ER, Hammoud SS (2023). Placental cell type deconvolution reveals that cell proportions drive preeclampsia gene expression differences. Commun Biol.

[80] Barrozo ER, Aagaard KM (2022). Human placental biology at single-cell resolution: a contemporaneous review. BJOG.

[81] Vasconcelos S, Canicais C, Chuva de Sousa Lopes SM, Marques CJ, Doria S (2023). The role of DNA hydroxymethylation and TET enzymes in placental development and pregnancy outcome. Clin Epigenet.

[82] Paauw ND, Lely AT, Joles JA, Franx A, Nikkels PG, Mokry M (2018). H3K27 acetylation and gene expression analysis reveals differences in placental chromatin activity in fetal growth restriction. Clin Epigenet.

