# Supplementary material for: Sex differences in DNA methylation across gestation: a large scale, cross-cohort, multi-tissue analysis
Source: Cell Mol Life Sci. 2024 Apr 10;81(1):177. doi: 10.1007/s00018-024-05208-0 (PMC11006734; doi:10.1007/s00018-024-05208-0)
Supplement: Supplementary file 1 — Supplementary file1 (PDF 134 KB) [file 18_2024_5208_MOESM1_ESM.pdf]

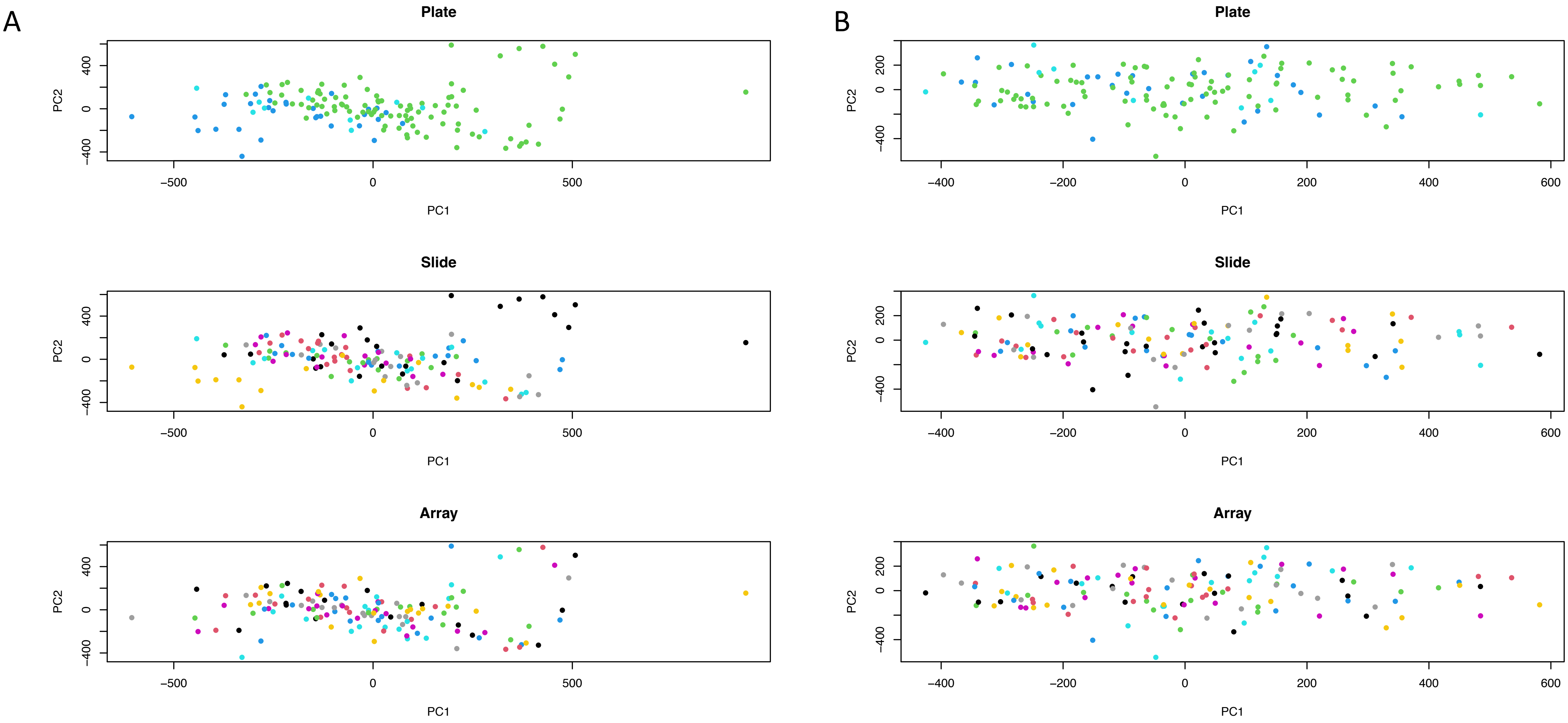

Figure S1_A: PCA plots illustrating Combat-correction in the BET-cohort
PC1 is plotted on the x-axis, PC2 is depicted on the y-axis. Different plates/slides and array positions are depicted by different colors. Panel A refers to variability before Combat-correction, panel B refers to variability after Combat-correction.
