# Supplementary material for: Sex differences in DNA methylation across gestation: a large scale, cross-cohort, multi-tissue analysis
Source: Cell Mol Life Sci. 2024 Apr 10;81(1):177. doi: 10.1007/s00018-024-05208-0 (PMC11006734; doi:10.1007/s00018-024-05208-0)
Supplement: Supplementary file 4 — Supplementary file4 (PDF 320 KB) [file 18_2024_5208_MOESM4_ESM.pdf]

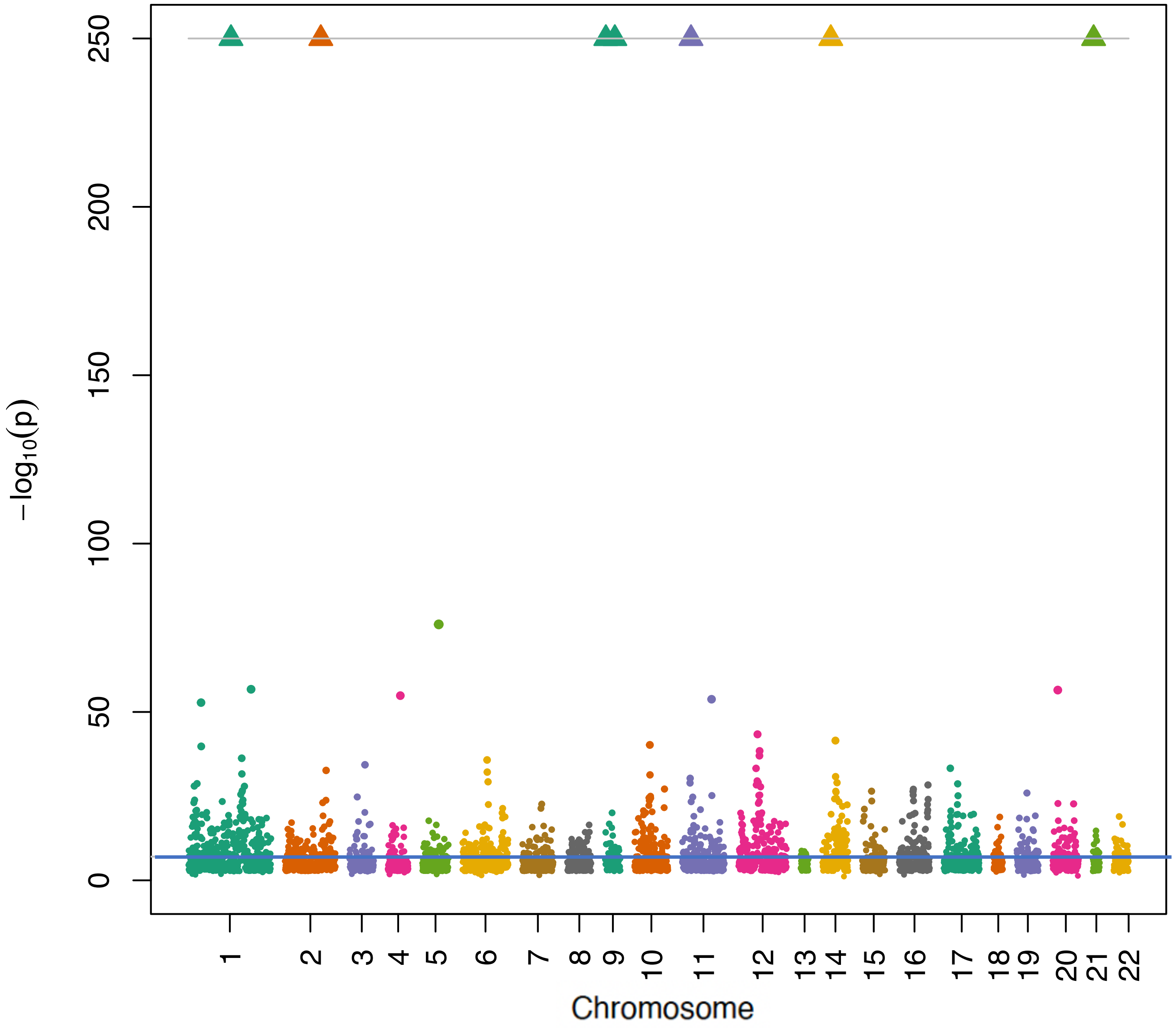

Figure S2: Manhattan plot of DMR-analysis of sex and DNAm in placenta.
The x-axis denotes the middle chromosomal position (based on hg19) of the DMR, the y-axis -log10(p-values). The blue line indicates the p-value-threshold for epigenome-wide significance. P-values below $1.0\text{x}10^{-250}$ were truncated and are depicted as triangles.
