# Supplementary material for: Sex differences in DNA methylation across gestation: a large scale, cross-cohort, multi-tissue analysis
Source: Cell Mol Life Sci. 2024 Apr 10;81(1):177. doi: 10.1007/s00018-024-05208-0 (PMC11006734; doi:10.1007/s00018-024-05208-0)
Supplement: Supplementary file 5 — Supplementary file5 (PDF 60 KB) [file 18_2024_5208_MOESM5_ESM.pdf]

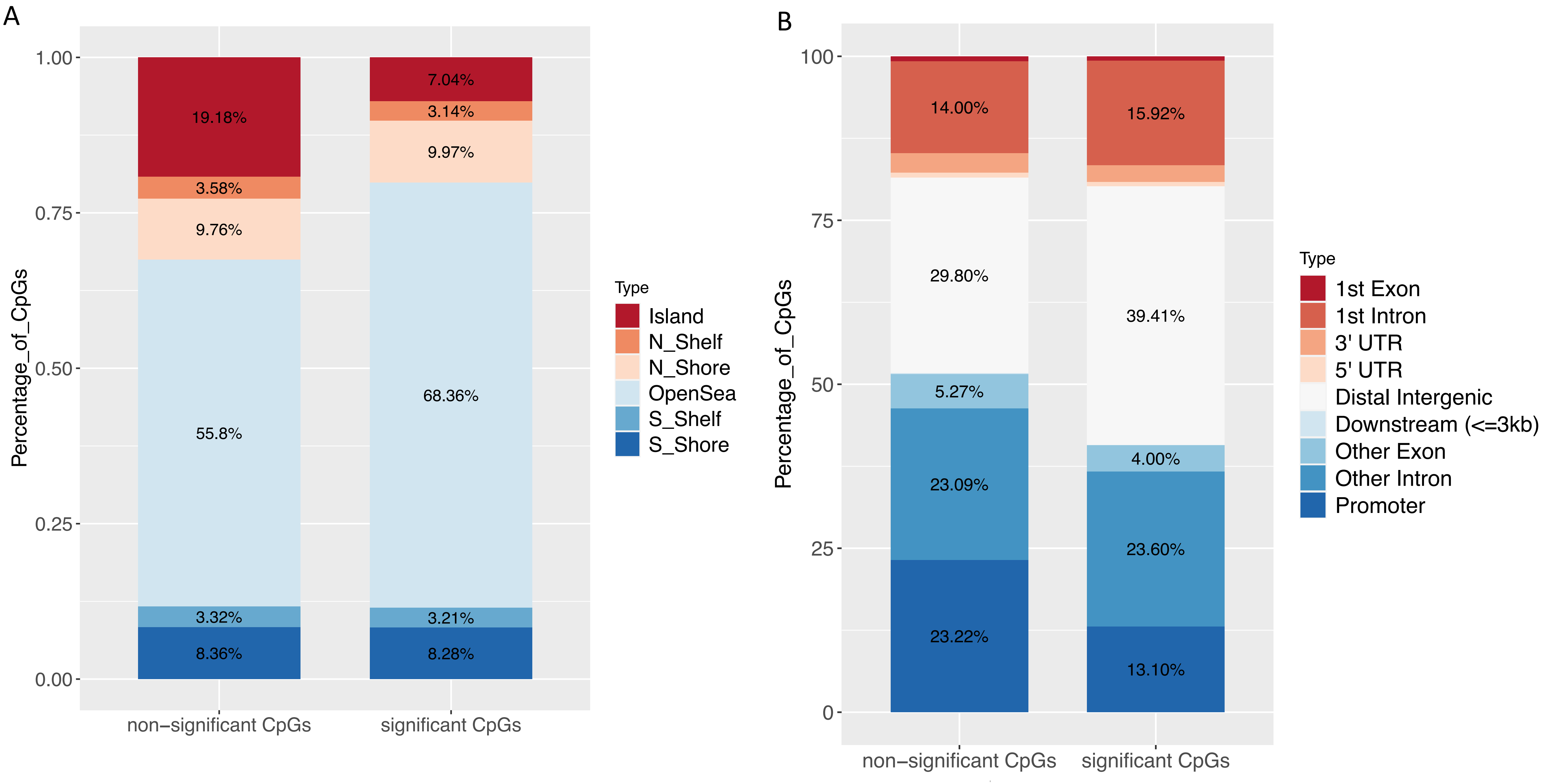

Figure S3: Position of epigenome-wide significant CpG-sites with relation to CpG-islands (A) and to genes (B).
